# Author Correction: Zero energy states clustering in an elemental nanowire coupled to a superconductor

**DOI:** 10.1038/s41467-022-35247-9

**Published:** 2022-11-30

**Authors:** Lauriane C. Contamin, Lucas Jarjat, William Legrand, Audrey Cottet, Takis Kontos, Matthieu R. Delbecq

**Affiliations:** Laboratoire de Physique de l’École normale supérieure, ENS, Université PSL, CNRS, Sorbonne Université, Université Paris Cité, F-75005 Paris, France

**Keywords:** Superconducting properties and materials, Electronic devices, Carbon nanotubes and fullerenes

Correction to: *Nature Communications* 10.1038/s41467-022-33960-z, published online 19 October 2022

The original version of this Article contained an error in the caption of Figure 3, which incorrectly read ‘Curves are PDF of Gaussian in solid black, Wigner surmise (WS) in orange and Tracy-Widom (TW) in blue, with solid lines for Gaussian orthogonal ensemble (GOE) and dashed lines for Gaussian unitary ensemble (GUE).’ The correct version states ‘Curves are PDF of Wigner surmise (WS) in orange and Tracy-Widom (TW) in blue, with solid lines for Gaussian orthogonal ensemble (GOE) and dashed lines for Gaussian unitary ensemble (GUE).’ This has been corrected in both the PDF and HTML versions of the Article.

