# Supplementary material for: Zero energy states clustering in an elemental nanowire coupled to a superconductor
Source: Nat Commun. 2022 Oct 19;13:6188. doi: 10.1038/s41467-022-33960-z (PMC9581951; doi:10.1038/s41467-022-33960-z)
Supplement: Supplementary file 1 — Supplementary Information [file 41467_2022_33960_MOESM1_ESM.pdf]

Supplementary Information for “Zero energy states clustering in an elemental nanowire coupled to a superconductor”

SUPPLEMENTARY DISCUSSION 1: CONTROL JUNCTIONS FOR NB/PD CHARACTERIZATION

The proximitized BCS DOS of the Nb/Pd electrode is characterized by doing Al/AlO$_x$/Pd/Nb junctions tunnel measurements in a cryostat at 300 mK. Differential conductance G traces normalized by the differential conductance trace in the normal state G_N are shown in Supplementary Figure 1 under application of an in-plane magnetic field (a,c) and perpendicular magnetic field (b,d). For each set, we show the raw data in panels (a,b) and data with a moving window average over 3 points in panels (c,d). The averaged curves allows to observe the transition of Al to normal through a discrete diminution of the gap width. The value of the superconducting gap $\Delta_{\mathrm{j}}^*(B)$ shown in Fig. 1c of the main text is extracted from each curve by fitting to the BCS DOS formula

$$\frac{G_S}{G_N} = \left| \Re\left(\frac{\epsilon + i\eta}{\sqrt{(\epsilon + i\eta)^2 - \Delta^2}} \right) \right|, \tag{1}$$

where η is the Dynes parameter which reflects the broadening due to finite lifetime of the quasiparticles in the superconductor. Fits of the data with eq .(1) in the perpendicular magnetic field situation are shown as dashed lines in Supplementary Figure 1d. As an example, for $B_\perp = 1$ T, we find $\eta = 0.16$ meV and $\Delta = 0.19$ meV and for $B_\perp = 2$ T, we find $\eta = 0.17$ meV and $\Delta = 0.12$ meV.

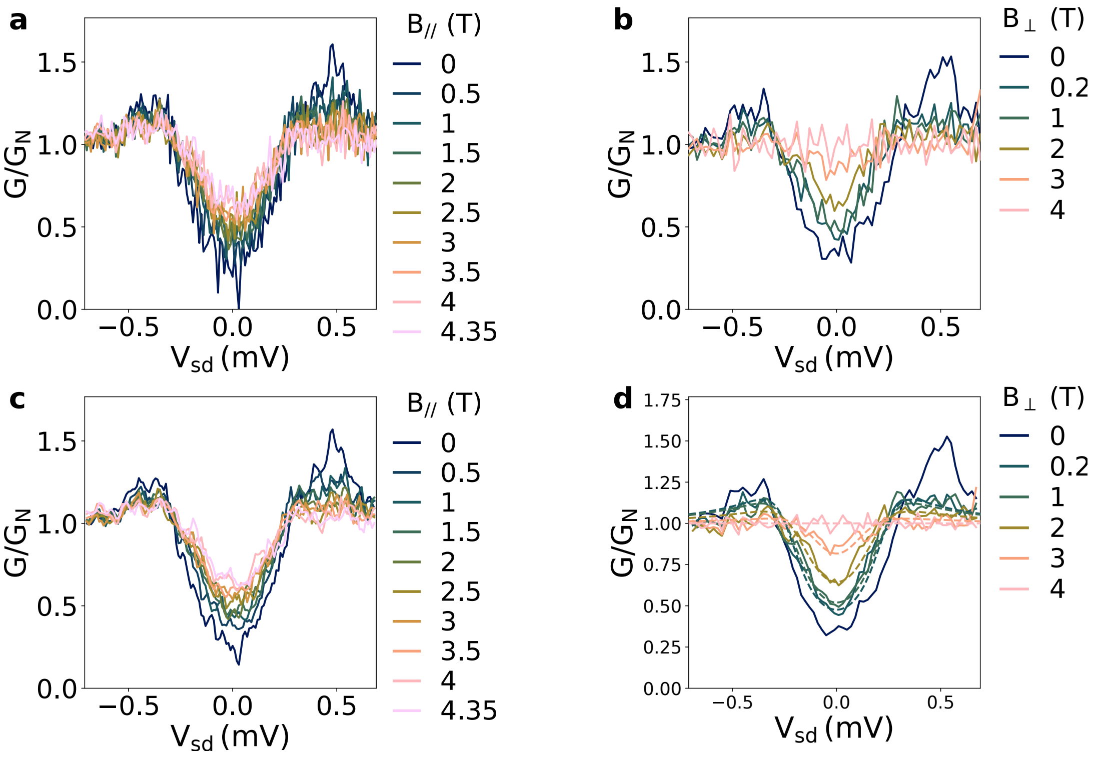

Supplementary Figure 1. **Al/AlO$_x$/Pd/Nb junction DOS.** Normalized conductance G/G_{N} with G_{N} the conductance in the normal state, at $B_\perp = 4$ T. Versus in-plane magnetic field (raw data **a** and with moving window averaging **c**) and perpendicular magnetic field (raw data **b** and with moving window averaging **d**). Dashed lines in panel **d** are fits to the data using eq .(1).

SUPPLEMENTARY DISCUSSION 2: MODEL SELECTION FOR THE Δ_{g} DISTRIBUTIONS

We analyze quantitatively the agreement between the different RMT predicted universal distributions and the histograms of Δ_{g} presented in Fig. 3 of the main text. For this we consider the Bayesian Information Criterion (BIC) generally considered the most conservative criterion for model selection. It is defined as

$$BIC = N \log(\chi^2/N) + \log(N) N_{\mathrm{param}}, \tag{2}$$

with N the number of data points, N_{param} the number of variable parameters and $\chi^2 = \sum_i^N r_i^2$ with r_i the residual for data point i. Accounting for uncertainty on data points, the residuals are defined as $r_i = (y_i - m_i)/e_i$ with y_i data point i, m_i the model value at point i and e_i the uncertainty on data point i. We note here that as there are no variable parameters to our models (Wigner surmise and Tracy-Widom) due to their universality, the BIC is equal to the Akaike Information Criterion (AIC), and comparing BIC in our case is equivalent to comparing χ^2 due to the monotony of the logarithm function. The lowest BIC indicates the model that accounts the best for the data. Applying eq. (2) to the data and models of Fig. 3 of the main text, we find the results presented in Supplementary Table 1. We find for B=0 T that BIC(WS$_1$) < BIC(TW$_1$) < BIC(WS$_2$) and for B=5 T that BIC(WS$_2$) < BIC(WS$_1$) < BIC(TW$_2$), where the index is the Wigner-Dyson index β with the correspondence $\beta = 1$ for GOE and $\beta = 2$ for GUE. This analysis quantitatively shows that our data is best described by the WS distribution in the GOE ensemble at $B = 0$ T, when time reversal symmetry is present, while the data at $B = 5$ T is best described by WS distribution in the GUE, corresponding to time reversal symmetry breaking and is thus fully consistent with the expected crossover from GOE to GUE predicted by RMT. It is important to note that inter datasets BIC values comparison has no meaning because BIC can be used to compare estimated models only when the numerical values are identical for all models being compared.

B (T)	GOE		GUE	
	WS$_1$	TW$_1$	WS$_2$	TW$_2$
0	-0.3	4.0	12.2	
5	12.5		12.0	16.4

Supplementary Table 1. BIC values for Wigner surmise (WS) and Tracy-Widom (TW) distributions in GOE (index 1) and GUE (index 2) for the two different datasets at $B = 0$ T and $B = 5$ T.

SUPPLEMENTARY DISCUSSION 3: EXTRACTION OF THE DEVICE ADDITION SPECTRUM

In this section we detail how the addition spectrum shown in Fig. 4d of the main text is constructed. We performed conductance measurement as a function of V_{g} and B at zero bias $V_{\mathrm{sd}} = 0$ mV. The signal-to-noise ratio (SNR) of the charge degeneracy resonance measurement (examples in Fig. 2(c,f) of the main text) is relatively low due to the small residual DOS, so that we identified 9 consecutive ground states that could be tracked with varying B. The low SNR imposed long averaging times and to perform the measurement the fastest possible to prevent any drift of charge jumps, we iteratively tracked each resonance as we sweep B from 5T to 0T, as shown in Supplementary Figure 2(a,b). The track of one of the states was lost during the procedure. Position of each peak $V_{\mathrm{g}}(n, B)$, with n indexing the peak, is extracted by fitting each peak with a Lorentzian. The resulting values are shown in Supplementary Figure 2(c) with the same labels as in the main text. Then $V_{\mathrm{g}}(n, B)$ is converted to energy by applying the capacitive leverarm $\alpha = C_{\mathrm{g}}/C_{\Sigma}$ conversion with C_{g} the capacitance between the CNT and the gate and C_{Σ} the total capacitance of the CNT to the gates. The leverarms are extracted from the slopes of the Coulomb diamonds of Fig. 2a of the main text. The construction of the addition spectrum requires to subtract the charging energy $E_c = e^2/C_{\Sigma}$ between consecutive levels, as has been done in other works (see refs [1, 2] for example). Here an important point is that the leverarm α fluctuates from one Coulomb diamond to the next, with values $\alpha \approx \{65, 60, 68, 60, 60, 66, 65, 66\} \pm 5$ meV/V for the states shown in Supplementary Figure 2(c). These fluctuations arise from fluctuations of C_{g} and C_{Σ} due to fluctuations of the electronic wave function with n. The fluctuations of the charging energy E_c is predicted by RMT [3, 4]) and is consistent the presence of mesoscopic fluctuations in the device. Therefore it is not possible to subtract a single charging energy value between the levels energy to construct the addition spectrum. Instead, the spectrum is constructed by matching the low magnetic field spectrum to the canonical CNT spectrum shown in Fig. 4a of the main text and as done in refs [1, 2]. In addition, in our situation there are many level crossings, due to the small level spacing, which adds many points of reference to connect the different levels together, as indicated by the dashed lines in Fig. 4d of the main text. Finally, the addition spectrum parameters (level spacing δ and $\sqrt{\Delta_{\mathrm{SO}}^2 + \Delta_{\mathrm{KK'}}^2}$) are further validated a posteriori by the agreement between the subgap states dispersion in B and the calculated excited spectrum shown in Fig. 5a of the main text.

SUPPLEMENTARY DISCUSSION 4: SPECTRUM OF A CNT NANOWIRE

The theoretical spectrum calculated with the extracted experimental parameters is shown in Supplementary Figure 3. We use $\delta = 325$ µeV from Fig. 4d of the main text, $g_{\mathrm{orb}} = 4.8$ from Raman spectroscopic characterization and

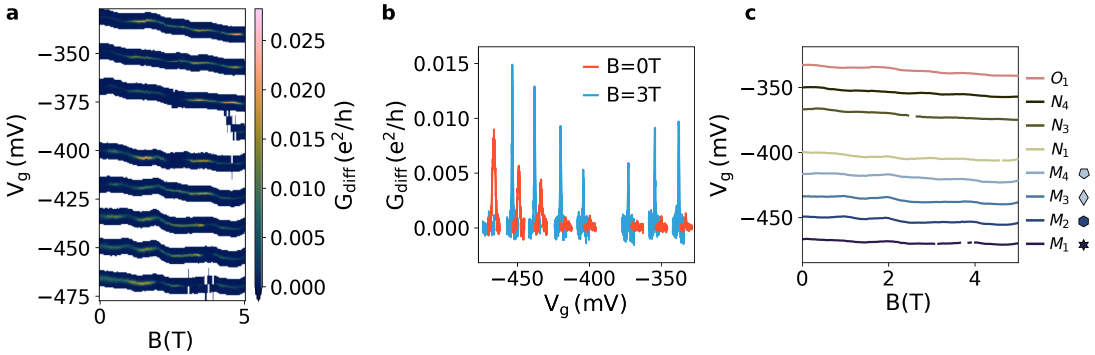

Supplementary Figure 2. **Charge ground state dispersion.** **a** Differential conductance $G_{\rm diff}$ as a function of B and $V_{\rm g}$ at zero bias $V_{\rm sd} = 0$ mV for 9 consecutive charge states. **b** Cuts at $B = 0$ T and $B = 3$ T extracted from **b**. **c** Positions of the peaks of **a** as a function of B with labels from the the main text.

$\theta = 14°$, the angle between the CNT and the external magnetic field (see Fig. 1a of the main text). The values of $\Delta_{\rm SO}$ and $\Delta_{\rm KK'}$ are constrained as $\sqrt{\Delta_{\rm SO}^2 + \Delta_{\rm KK'}^2} \approx 100$ μeV. There is thus a single fitting parameter, $\Delta_{\rm SO} = -40$ μeV (with $\Delta_{\rm KK'} = \sqrt{100^2 - \Delta_{\rm SO}^2} = 87$ μeV), to adjust the magneto-spectrum of Fig. 5a of the main text. The finite value of the inter-valley coupling is not incompatible with having a clean CNT. The coupling to the superconducting contact breaks rotational symmetry in the CNT, and therefore leads to finite inter-valley coupling. Second the inter-valley coupling occurs within a given orbital (or transverse subband). It happens only once, at a magnetic field $B_{\rm KK'} = \pm\sqrt{\Delta_{\rm SO}^2 + \Delta_{\rm KK'}^2}/(2g\mu_B) \approx \pm 0.18$ T with our parameters. Therefore for all the magnetic field range beyond $B_{\rm KK'}$, the spectrum behaves as with having no band coupling, justifying that our observations are in the no inter-subband coupling regime.

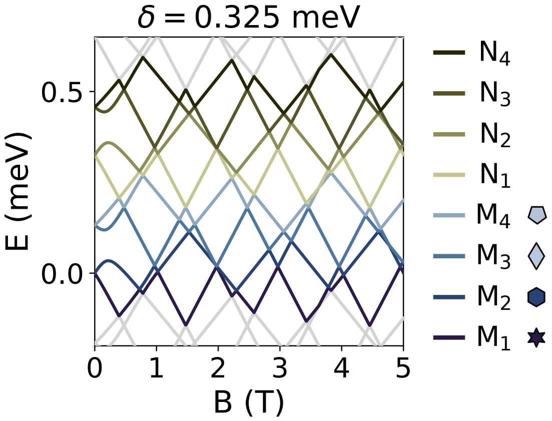

Supplementary Figure 3. **Theoretical spectrum of our NW.** Calculated addition spectrum of a CNT with the parameters $\delta = 325$ μeV, $g_{\rm orb} = 4.7$, $\theta = 14°$ extracted from experimental data. Fit parameter to adjust the magneto-spectrum of Fig. 5a of the main text is $\Delta_{\rm SO} = -40$ μeV and $\Delta_{\rm KK'} = \sqrt{100^2 - \Delta_{\rm SO}^2} = 92$ μeV.

The global negative slope observed for all levels in Fig. 4d of the main text is not present in the canonical CNT spectrum and its origin remains unclear. It could be an effect of superconductivity although it would need to be investigated theoretically. However it would not affect the calculation of the excited states spectrum as it consists in the energy difference between consecutive states so that the slope cancels out. We would like also to briefly discuss the difference between the measured addition spectrum of Fig. 4d of the main text and the excited spectrum that is observed in the magneto-spectroscopy (Fig. 5a of the main text) and calculated in Supplementary Figure 3. Although one could expect them to be identical, it is experimentally established that this holds true at weak magnetic field only. For example, in ref. [1], only the two-electron ground state is identical to the first excited state of the single-electron ground state on the measured magnetic field range, but the agreement fails already for the three-electron ground state (within a small magnetic field range, below 0.2 T). Since we are in a many electron ground state regime, and at significantly higher magnetic fields, it is therefore not surprising that we observe a discrepancy between the addition and excitation magneto-spectra. The addition spectrum at low magnetic field still allows us to extract the relevant

parameters to calculate the excited spectrum.

SUPPLEMENTARY DISCUSSION 5: MAGNETO-SPECTROSCOPY OF CHARGE GROUND STATE "M_2" AND "M_4"

The magneto-spectroscopy of the charge ground state M_3 labeled by a diamond is discussed in the main text, with a remarkable agreement between the sub-gap states and the calculated spectrum using the parameters of Supplementary Figure 3. This interpretation is further strengthened by investigating the magneto-spectroscopy of the previous and next charge ground states, M_2 and M_4, labeled by an hexagon and a down-pointing pentagon respectively, as displayed in Supplementary Figure 4. The spectrum calculated with the same parameters as for state M_3 shows again a good agreement with the data with several diamond-like features being shifted in B between the data and the model as shown in Supplementary Figure 4a,c. For charge state M_2, changing slightly one parameter, the level spacing δ from 325 µeV to 350 µeV yields the spectrum shown in Supplementary Figure 4b which matches very well the experimental data. For charge state M_4, changing δ to 320 µeV and g_{orb} from 4.8 to 5 yields the spectrum shown in Supplementary Figure 4d which matches again very well the experimental data. It is reasonable that the small variations of these parameters, even within a single shell, are due to mesoscopic fluctuations similar to the one giving rise to fluctuations of the minigap. In any case, the parameters small changes are reasonable within experimental uncertainties and essentially validates our model.

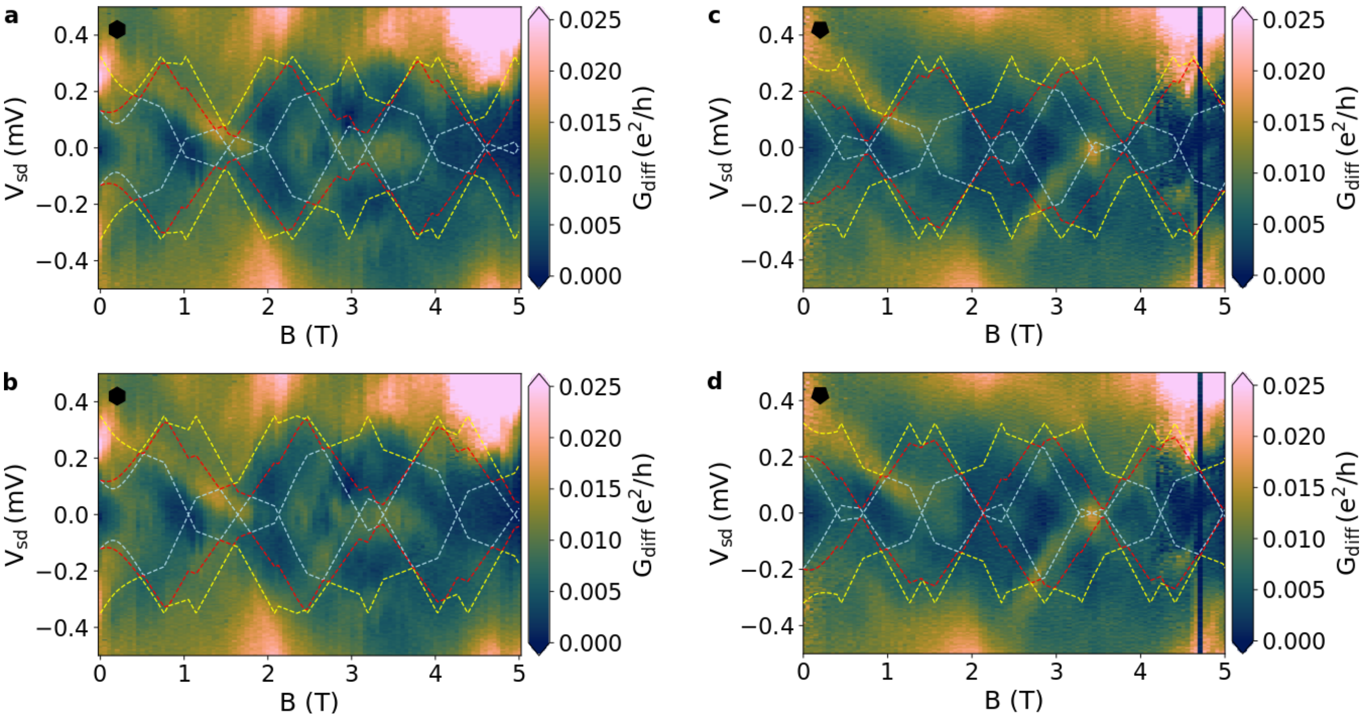

Supplementary Figure 4. **Magneto-spectroscopy of charge states "M_2" and "M_4". a.** and **b.** Differential conductance G_{diff} as a function of bias voltage V_{sd} and magnetic field B at the Coulomb diamond degeneracy point indicated by a hexagon symbol around $V_{\mathrm{g}} \approx -382$ mV in Fig. 2a of the main text. The ground state corresponds to state M_2 and the first 3 excited states to M_3 (dashed blue), M_4 (dashed red) and N_1 (dashed yellow). **a.** The excited states are calculated with the parameters of Supplementary Figure 3. **b.** The excited states are calculated with the parameters of Supplementary Figure 3 with δ changed to 350 µeV. **c.** and **d.** Same for the Coulomb diamond degeneracy point indicated by a down-pointing pentagon symbol around $V_{\mathrm{g}} \approx -347$ mV in Fig. 2a of the main text. The ground state corresponds to state M_4 and the first 3 excited states to N_1 (dashed blue), N_2 (dashed red) and N_3 (dashed yellow). **c.** The excited states are calculated with the parameters of Supplementary Figure 3. **d.** The excited states are calculated with the parameters of Supplementary Figure 3 with δ changed to 320 µeV and g_{orb} changed to 5.

SUPPLEMENTARY DISCUSSION 6: MAGNETO-SPECTROSCOPY OF VARIOUS CHARGE GROUND STATES

The magneto-spectroscopy data in the $B - V_{\mathrm{sd}}$ plane of 16 charge ground states is shown in Supplementary Figure 5 and Supplementary Figure 6. Each map is labeled by a symbol displayed in Fig. 2a of the main text that specify charge ground state is considered. Open symbols indicate magneto-spectroscopies where no clear ZBCP was observed while filled symbols indicate ones where one or more ZBCP are observed. We observe roughly three types of maps in terms of global behaviour or features. When compared to each others, maps of Supplementary Figure 5a to d show relatively large subgap states; maps of Supplementary Figure 5e to h show few and low amplitude subgap states, rendering an almost empty gap; maps of Supplementary Figure 6a to h show thinner subgap states than the first group with again an amplitude comparable to quasiparticle peaks. Such global changes can be attributed to slow change of parameters like the coupling to the leads which are affected by the gate potential V_{g} and the electron wave function. We identify 13 maps out of 16 where at least one ZBCP is observed, showing their ubiquity in our device, in line with the interpretation based on clustering of zero energy states due to mesoscopic fluctuations.

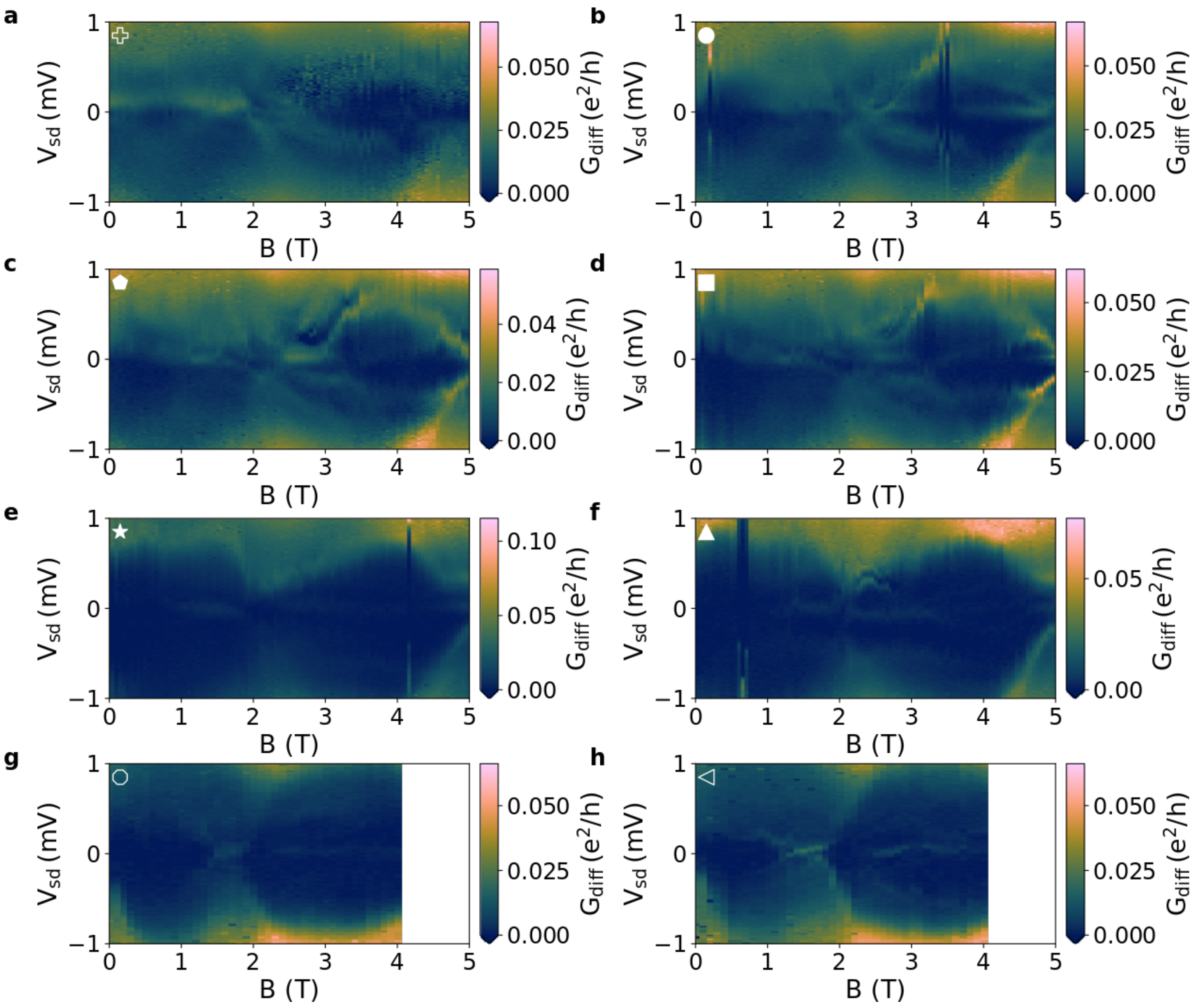

Supplementary Figure 5. **Magneto-spectroscopy of various charge ground states (1/2).** The corresponding charge ground states are identified by the symbols in the top left corner of each panel, referring to symbols displayed in Fig. 2a of the main text.

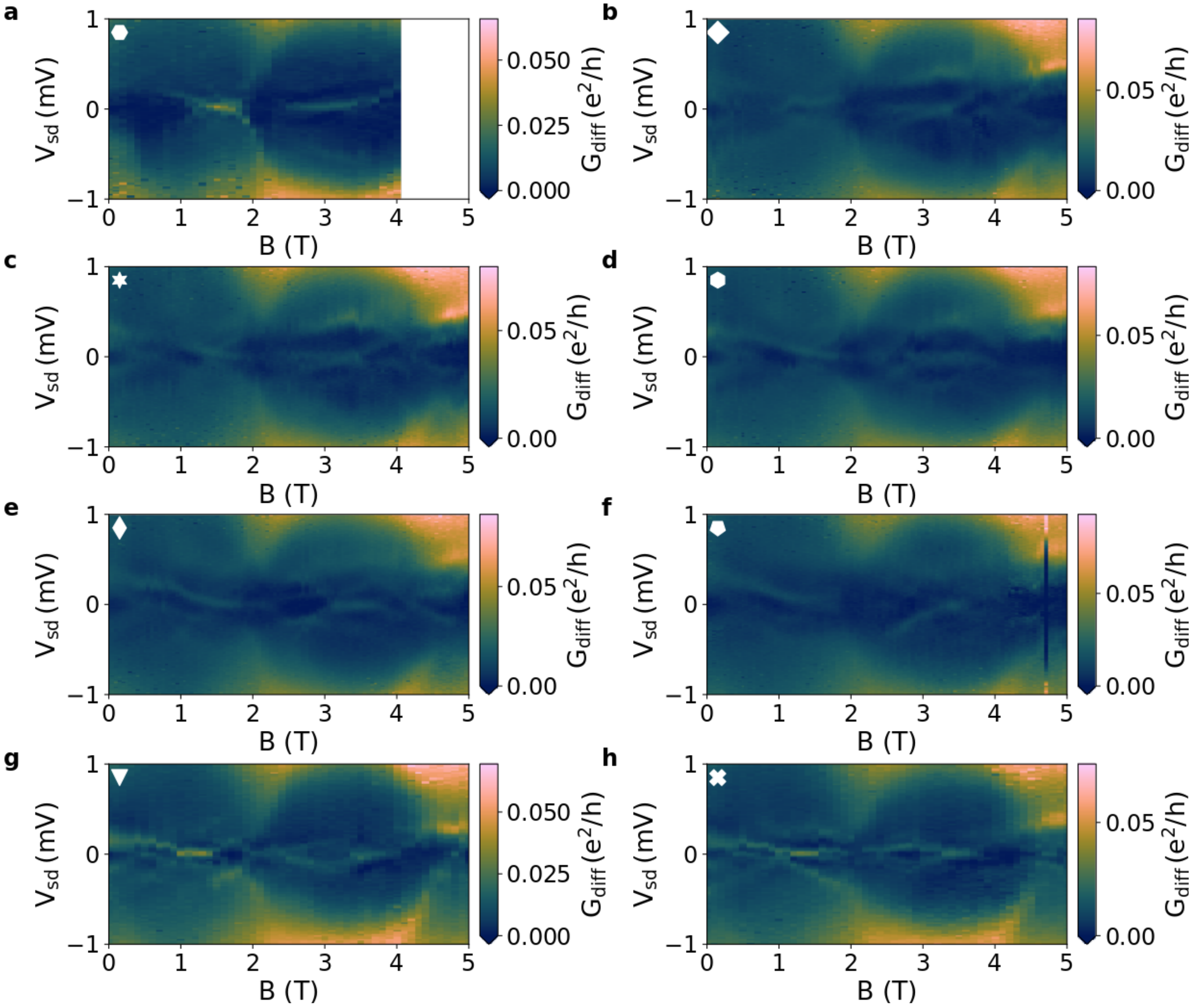

Supplementary Figure 6. **Magneto-spectroscopy of various charge ground states (2/2).** The corresponding charge ground states are identified by the symbols in the top left corner of each panel, referring to symbols displayed in Fig. 2a of the main text.

[1] F. Kuemmeth, S. Ilani, D. C. Ralph, and P. L. McEuen, Nature **452**, 448 (2008).
[2] G. Steele, F. Pei, E. Laird, J. Jol, H. Meerwaldt, and L. Kouwenhoven, Nature Communications **4**, 1573 (2013).
[3] C. W. J. Beenakker, Reviews of Modern Physics **69**, 85 (1997).
[4] Y. Alhassid, Reviews of Modern Physics **72**, 895 (2000).
